# Optimization of synthesis constituents of slow-release zinc fertilizers using response surface methodology

**DOI:** 10.1371/journal.pone.0345863

**Published:** 2026-04-29

**Authors:** Ava Mohrazi, Reza Ghasemi-Fasaei

**Affiliations:** Department of Soil Science, School of Agriculture, Shiraz University, Shiraz, Iran; Rodale Institute, UNITED STATES OF AMERICA

## Abstract

Zinc (Zn) deficiency in calcareous soils is a global issue that affects agricultural yields and threatens nutrient availability worldwide. The present study examined optimization of elements influencing synthesis of a slow-release fertilizer (SRF) of Zn and compared it with commercial ZnSO_4_ fertilizer. In this study, response surface methodology (RSM) optimization technique was used to reduce costs and time. Organic (pectin, biochar), inorganic (layer double hydroxide (LDH), layer double hydroxide (LDO)), and microbial (bacterial inoculation) treatments were utilized, leading to production of 12 slow-release fertilizers. Ultimately, optimization and modeling were carried out. Based on RSM findings, three fertilizers exhibiting highest Zn levels and greatest desirability (SRF-LDH, SRF-LDO-bacterial, SRF-biochar-LDO-bacterial) were recognized as most efficient fertilizers. LDH increased Zn concentration by 75% and 50% compared to LDO and biochar, respectively. Based on the results, biochar increased Zn concentration by 40% compared to pectin. Additionally, combining biochar with LDH increased Zn concentration by 60% compared to biochar alone. The highest Zn concentration of SRFs (93.48 mg g ^−1^) was related to run 3 (organic material: 0, inorganic material: LDH, inoculation bacteria with fertilizer: 1 W/V), which is 3.9-fold higher than the lowest run (24.54 mg g ^−1^) (run 9: organic material: pectin, inorganic material: 0, inoculation bacteria with fertilizer: 0). In comparing all runs, where biochar is used instead of pectin in SRFs, the Zn concentration increased. The highest swelling ratio was related to SRF1 (4.4 g g -¹). The swelling ratio of SRF3 (LDO-biochar-bacterial) was 3.81 g g -¹, which was higher than that of ZnSO_4_ (2.41 g g -¹). The slow-release behavior of Zn from SRFs demonstrated three phases, comprising an initial phase of steady Zn release and a concluding phase of diminishing Zn release rates. First-order and Higuchi models were appropriate for characterizing slow-release nutrient processes of SRFs, suggesting external environment can affect gradual release of Zn from SRFs. Based on the RSM optimization results, three formulations—SRF-LDH, SRF-LDO-bacterial, and SRF-biochar-LDO-bacterial—were identified as the most efficient among the twelve synthesized SRFs, exhibiting the highest Zn loading and desirability values.

## Introduction

Zinc (Zn) is an essential micronutrient, and its deficiency is common in alkaline -calcareous soils [1]. It causes optimal production and metabolic functioning of plants, such as growth regulation and protection against diseases, among others. Most farmland in arid and semi-arid areas has low Zn bioavailability due to low organic matter, high pH, and calcium carbonate (CaCO_3_). Therefore, Zn deficiency is a common problem in agricultural products of these regions. Zn plays a vital role not only in plants but also in human health. Its deficiency in humans reduces learning ability, immune system function, and more. Although Zn is found in abundance in the human body, humans cannot store it, so they need to obtain it regularly from food. Therefore, adequate dietary Zn intake is essential to address nutrient deficiencies, making the use of Zn fertilizer crucial in agriculture [2].

Zinc sulfate (ZnSO_4_), zinc nitrate (Zn (NO_3_) _2_), zinc oxide (ZnO), and chelated Zn (Zn-EDTA) are known as commercial fertilizers that are extensively used for mitigating Zn deficiency in farmlands and improving crop production. If commercial fertilizers are excessively used over time, they can cause water pollution, soil degradation, greenhouse gas emissions, and an imbalance of nutrients in the soil (Zn prevents the uptake of iron (Fe) and copper (Cu) by plants) [3]. On the other hand, a disadvantage of commercial zinc fertilizers in alkaline soil is the rapid conversion of the soluble form (ZnSO_4_) to the insoluble form (Zn (OH)), which disrupts Zn uptake by plants. Therefore, farmers use excessive amounts of these fertilizers to prevent Zn deficiency, which increases costs and wastes energy [4].

To maximize crop yield, mitigate Zn deficiency, maintain human health, prevent soil degradation, and save money and energy, one of the key solutions is slow-release Zn from fertilizers. Slow-release fertilizers based on essential nutrients (SRF) are suggested as a promising, cost -effective, easy, and green technology to enhance essential nutrient uptake by plants and mitigate soil and water pollution. In fact, the main mechanism of these fertilizers is releasing macro and micronutrients slowly to meet plant needs during crop growth [5].

However, it should be noted that the effectiveness of slow-release fertilizers depends on several factors, such as the appropriate dosage, the correct timing of application, the proper method, and the raw materials used. The incorporation of nutrients into porous materials is a significant method for producing slow-release fertilizers (SRFs). The main characteristics of these materials are their insolubility, low cost, abundant availability, environmental compatibility, biodegradability, and high porosity [6].

SRFs can be generally categorized into organic and inorganic forms. Inorganic forms include clay minerals such as zeolite, bentonite, and synthetic forms of clay, including layered double hydroxides (LDH) and their calcined form, layered double oxides (LDO). The partial replacement of certain divalent cations with trivalent ions leads to the formation of positively charged layers within LDH. Consequently, LDH exhibits a strong affinity for cationic groups, including hydroxide, selenate, carbonate, and sulfate, which aids in maintaining charge equilibrium [7]. Furthermore, LDH is characterized by its remarkable stability, substantial loading capacity, effective cation exchange capacity, and favorable surface-to-volume ratio, rendering it a valuable option as slow-release carriers for cationic nutrients Actually, it has the ability to gently release the interlayer cations of LDHs and quickly release nutritional ions from its exterior surfaces. In addition, LDH is employed to regulate the release rate of essential nutrients such as Zn and Se. Several reports on the release of essential nutrients from LDH are available in the literature [8]. Shafigh [9] reported that Zn-Fe-Mg-LDH released Zn from LDH, which helped decrease Zn deficiency in soil and enhanced crop production. However, the kinetic results of Zn release from LDH showed that Zn-LDH was a good choice for use as SRFs. In addition, organic forms are divided into two categories: natural (plant residues and animal manure) and synthetic (cellulose, lignin, biochar, pectin).

Biochar, characterized by its porous nature, has the potential to enhance soil porosity and improve the soil’s ability to retain water, thereby allowing for greater nutrient availability for plants. Moreover, biochar is noted for its significant biological stability and its role in promoting carbon sequestration within the soil [6]. In contrast to conventional fertilizers, biochar can be employed in the formulation of biochar-based fertilizers. Additionally, biochar serves as an effective medium for the adsorption of heavy metals (HMs). Some studies have proven that biochar has the potential to release nutrients, but its efficiency depends on its characterization, preparation conditions, and the pH of the environment. Among these factors, biochar characterization is more important than the others. The characterization of biochar, such as porosity, specific surface area (SSA), and functional groups, can be enhanced through modification [5]. Nevertheless, investigations indicate that unmodified biochar has a limited capacity for adsorption, underscoring the necessity for modifications to enhance its performance. Thus, the modification of biochar is necessary for its use as a slow-release fertilizer. One way to modify and improve the properties of biochar is to connect this organic compound to LDH. This connection enhances the stability of biochar and increases porosity, thus improving its composition for producing SRFs [10].

In addition to the above, the use of growth-promoting bacteria increases the efficiency of fertilizer application and improves plant performance. These bacteria stimulate the production of hormones in the plant and enhance the solubility of insoluble compounds, which increases the availability of nutrients. They also reduce ethylene production in stressed plants by producing the hormone ACC deaminase. Bacterial inoculation of LDH resulted in increased zinc bioavailability for plants, as demonstrated by Shafigh et al [11]. The bacterial species *Pseudomonas, Agrobacterium, and Bacillus* are microorganisms that improve nutrient solubility and make fertilizer ingredients easier to release.

According to researchers’ studies, various factors affect the quality of SRFs. Therefore, considering the importance of production, cost savings, and energy conservation, statistical optimization methods should be used to synthesize these fertilizers. One such statistical method is the response surface method (RSM) [12]. In addition to being economical, this method offers higher accuracy than other statistical methods, such as Taguchi [13].

According to our knowledge, no study has been conducted in the field of optimizing SRFs synthesis. Thus, considering the need to enhance researchers’ understanding of SRFs, the present study was carried out. The main objective of this research was to optimize the types and combinations of constituents effective in the synthesis of slow-release Zn fertilizer using organic raw materials (biochar and pectin), besides mineral materials (LDH and LDO), along with bacterial inoculation.

## Materials and Methods

### Materials

All chemical reagents and Licorice pomace used in the present research were purchased from Sigma Co. and Fars Osareh Iranian factory.

### Experimental Design

To maximize the synthesis of Zn-SRFs, three independent factors were employed in this study: the ratio of bacterial inoculation with fertilizer, inorganic minerals (LDH and LDO), and organic materials (biochar and pectin). To determine the optimal Zn-SRFs, the RSM model was used to organize the experiment, optimize settings, and construct the link between factors and response. The experimental design in this study was the Box-Behnken design (BBD), a second-order model based on a three-level incomplete factorial design with 3 replicates in central point. Also coded by actual. Table 1 displays the experiment’s design and independent variables. A 2F1 model was used in this study to express the connection between the independent variables and response. (Eq. 1).

**Table 1:** The experimental factors and BBD design matrix for the independent variables.

variable	unite		Range	
		−1	0	1
A: organic material type	–	0	Pectin	biochar
B: inorganic material type	–	0	LDO**	LDH*
C: Bacterial inoculation ratio with fertilizer	W/V	0	1	2
**Run**	**A**	**B**	**C**	
1	0	0	0	
2	biochar	0	1	
3	0	LDH	1	
4	biochar	LDH	1	
5	0	LDO	0	
6	biochar	LDO	0	
7	0	LDO	2	
8	biochar	LDO	2	
9	pectin	0	0	
10	pectin	LDH	0	
11	pectin	0	2	
12	pectin	LDH	2	
13	pectin	LDO	1	
14	pectin	LDO	1	
15	pectin	LDO	1	

*layer double hydroxide (LDH), **layer double oxide (LDO)


$$\text{Y}={\text{b}}_{\text{0}}+{\text{b}}_{\text{1}}\text{A}+{\text{b}}_{\text{2}}\text{B}+{\text{b}}_{\text{3}}\text{C}+{\text{b}}_{\text{12}}\text{AB}+{\text{b}}_{\text{13}}\text{AC}+{\text{b}}_{\text{23}}\text{BC}$$ (1)

Where Y represents the Zn concentration of SRF, A, B, and C define organic materials, inorganic materials, and the bacterial (*Bacillus subtilis*) inoculation ratio with fertilizer, respectively. AB, AC, and BC indicate the interaction of the independent variables. b₀ is the intercept coefficient, while b₁, b₂, and b₃ are the linear coefficients of the model. b₁₂, b₁₃, and b₂₃ are the interaction coefficients between the factors. b₁₁, b₂₂, and b₃₃ are the second-order coefficients of the model.

### Preparation of Slow Release Fertilizers

The preparation procedures for biochar and pectin: Initially, to synthesize organic materials such as biochar and pectin, it is necessary to prepare a raw material like licorice pomace. After preparation, the pomace was washed, dried, ground, and finally placed in a furnace at 600 °C for 2 h to produce biochar. On the other hand, to synthesize pectin, 60 g of licorice pomace was initially mixed with 600 ml of water, and its acidity was adjusted to 4 using HCl (2N), and then placed in a microwave for 2 min. After reaching room temperature, it was centrifuged, passed through a paper filter number 1, and finally placed in an oven at 65 °C for 24 h [14; 15].

The preparation procedures for LDH and LDO: A simple and inexpensive co-precipitation method was used to prepare inorganic materials such as LDH and LDO. Briefly, magnesium chloride (MgCl₂) and aluminum chloride (AlCl₃) (the source and purity of magnesium chloride (MgCl₂) and aluminum chloride (AlCl₃) are Mineral compounds that is purchased from Sigma company with 98% purity) were first mixed in distilled water with a cation ratio of 4:1 and stirred for 30 min. Then, sodium hydroxide (2 M) was added dropwise to increase the pH to 9. The mixture was kept at 65 °C for 24 h, after which the suspension was centrifuged, the supernatant was discarded, and the remaining compounds were washed with distilled water to obtain LDH. Subsequently, the LDH was calcined in a furnace at 600 °C for 3 h to produce LDO. In fact, LDO is a layered metal oxide obtained by calcining LDH [16].

The preparation procedures for organic matter – crosslinked inorganic matter: Based on Table 1, it is necessary to synthesize composite fertilizers such as biochar/LDH, biochar/LDO, pectin/LDH, and pectin/LDO. Inorganic material was mixed with organic material in a 1:1 (w/w) ratio to synthesize the composite fertilizer. Organic material (pectin or biochar) was added to 20 ml of distilled water and sonicated for 25 min to break up large solid particles of biochar or pectin [17]. After that, inorganic material (LDH or LDO) was added to the mixture to form the fertilizer compounds, and the pH was adjusted to approximately 10–11 with NaOH (2 M). Finally, it was stirred for 24 h, filtered, and washed 6 times [18].

### Fertilizer Inoculation with Bacteria

To synthesize the inoculated form of these fertilizers with bacteria, the SRFs were inoculated with *Bacillus subtilis* in a ratio of 1:2 and 1:3 (w/v) [19]. For inoculation, the SRFs were sprayed with bacteria at the stated ratios and kept at a temperature of 15–25 °C for 8 h [19,20]. Bacillus subtilis bacteria were in the lyophilized powdered form in the concentration of 1 × 109 CFU g^-1^. 1 gr of lyophilize bacteria dissolved in 100 ml distilled water then spray to SRFs.

### Fertilizer enrichment with Zn

Finally, to synthesize Zn-SRFs, the prepared bases were added to 20 ml of a 7% solution containing Zn and shaken for 48 h at 65 °C. Then, the mixture was centrifuged, and the slow-release fertilizer was separated from the solution and dried for 24 h at 65 °C.

### Zn concentration in SRFs

To measure zinc concentration, 1 g of SRFs was first digested with a 4:1 solution of perchloric acid and nitric acid [21]. After measuring the concentration of all fertilizers, modeling and optimization were performed using the RSM method. Finally, 3 optimized SRFs were selected, and their characteristics were assessed.

### Characterization of optimal SRFs

After synthesizing the SRFs, the structural properties and morphophysiological characteristics, including the surface area and distribution of its pores, specific surface area, and functional groups, were measured using Brunauer-Emmett-Teller (BET), scanning electron microscopy-energy dispersive X-ray analysis (SEM-EDX), and Fourier-transform infrared spectroscopy (FT-IR) techniques, respectively.

### Evaluation of swelling ratio, water – holding capacities, and stability test

2.4.1. Swelling Test: One gram of all treatments, including three optimal SRFs and ZnSO_4_·7H2O as the control treatment, was placed in a tea bag and immersed in 200 mL of distilled water at room temperature (25 °C) [6]. The treatments were weighed at certain times (0 min, 1.5 h, 3 h, 6 h, 12 h, 24 h, and 48 h). The equation used for calculating the swelling ratio (SR) of SRFs was as follows:


$$\text{SR}=\frac{M_{a}-M_{b}}{M_{a}}$$ (2)

where M_a_ is the mass of SRFs after immersion at certain time, M_b_ is the mass of SRFs before immersion

The Korsmeyer-Peppas model (Eq. (3) and the pseudo-second-order (PSO) kinetic model (Eq. (4) were applied to fit the swelling rate of SRFs for investigating the swelling mechanisms


$$\text{The Korsmeyer }-\text{ Peppas model}:\frac{Q_{t}}{Q_{e}}=K_{KP}t_{n}$$ (3)

$$\text{The pseudo}-\text{second}-\text{order}:\frac{t}{Q_{t}}=\frac{\text{1}}{k_{\text{2}}\overset{\text{2}}{\underset{e}{Q}}}+\frac{\text{1}}{Q_{e}}t$$ (4)

The water absorbed mass at time t, the water absorbed mass at equilibrium time, the PSO swelling constant, the rate constant, and the diffusional exponent were denoted by Q_t_, Q_e_, k_2_, K_KP_, and n, respectively.

### Water-holding capacities

Tests of water-holding capacity were performed to assess the SRFs’ water-retention ratios. In short, 100 g of zeolite with 60% water content was mixed with 1 g of each treatment in a beaker, and the mixture was weighed (W_F_). Weights (W_N_) were recorded over a period of days (1, 2, 3, 4, and 5 days) while all treatments were carried out at room temperature (25 °C) [22]. The water-holding capacity ratio (WR %) was calculated using the following formula:


$$\text{WR}=\frac{M_{F}}{M_{N}}$$ (5)

where M_F_ and W_N_ represented the total mass of zeolite and SRFs on the first day and the certain day, respectively.

### Stability test

The impact of pH on the stability of SRFs was evaluated based on the previous method. Samples of 0.1 g of each dried SRF were placed into 50 mL of distilled water with a pH range of 3–11 and kept at room temperature for one day [23]. The weight of each SRF was measured before (Mi) and after (Mf) 24 h, and the value of loss percentage (DL%) was calculated using the following formula (Eq. (5)):


DL%=(Mi−Mf)/Mi ×100 (6)

### Zn release behaviors and kinetics

The released percentage of Zn from the SRFs was studied. Briefly, 0.1 g of each SRF was placed in tea bags and soaked in 200 mL of distilled water. To detect the concentration of Zn, 3 mL of each solution at periods of 0, 1.5, 3, 6, 12, 24, 48 h was extracted from the beaker and measured using atomic absorption spectroscopy (AAS). The Zn release test results were fitted to four release kinetics, including zero-order, first-order, Higuchi, and Korsmeyer-Peppas models (Eqs. 6–9).


$$\text{The Zero }-\text{ order kinetics}:\text{ Y }={\text{K}}_{\text{0}}\text{t}$$ (7)

$$\text{The First }-\text{ order kinetics}:\text{ Y }=\text{1}-\text{ e}-K_{\text{1}}\text{t}$$ (8)

$$\begin{array}{c} \text{The Higuchi model}:\text{ Y }=K_{H}\text{t ½} \end{array}$$ (9)

$$\text{The Korsmeyer}-\text{Peppas model}:\text{ Y }=K_{KP}\text{tn}$$ (10)

In these equations, Y is the fertilizer release fraction at time t. t is the release time. KKP and n are the release constant and the release power, respectively. KH is the Higuchi dissolution constant. K_0_ and K_1_ are the release constants of the zero-order and first-order models, respectively. Each of these constants represents a specific behavior. For example, if the K_KP_ coefficient in The Korsmeyer −Peppas model, is high, it means that the Zn release rate is high; if it is low, it indicates that the Zn release rate is slow [24].

### Results and discussion

The influence of three independent variables, namely A, B, and C, on the Zn concentration of SRFs is shown in Table 2. Based on the RSM- box behnken design (BBD) model, a nonlinear 2F1 model was applied to the data through multiple regression analysis. Furthermore, the final model, following the variable selection process and the removal of factors that were not statistically significant (p > 0.05), is presented as follows (Eq. 10):

**Table 2:** box behnken design (BBD) matrix for the three independent variables, including the actual and predicted responses along with the percentage error.

Run	A	B	C	actual	predict	PE (%)
1	0	0	0	0	0	0
2	biochar	0	1	42.34	42.37	0.05
3	0	LDH*	1	93.48	97.46	4.25
4	biochar	LDH	1	31.2	32.15	2.84
5	0	LDO**	0	40.45	37.54	7.21
6	biochar	LDO	0	31.54	33.28	5.48
7	0	LDO	2	70.32	77.26	9.86
8	biochar	LDO	2	90.90	82.37	9.38
9	pectin	0	0	24.52	23.58	3.83
10	pectin	LDH	0	47.48	47.25	0.49
11	pectin	0	2	42.24	36.13	14.47
12	pectin	LDH	2	32.77	41.24	25
13	pectin	LDO	1	54.67	47.33	13.42
14	pectin	LDO	1	48.67	47.33	2.75
15	pectin	LDO	1	45.10	47.33	4.93

*layer double hydroxide (LDH), **layer double oxide (LDO)


Zn concentration of SRFs =+47.33 − 10.07A + 17.48B + 11.92C + 22.59AB +7.94AC +5.64BC (11)

The equation expressed in terms of coded factors allows for predictions regarding the response at specified levels of each factor. The coefficient’s sign indicates the nature of the relationship between the independent variable and the Zn concentration of SRFs.

A positive sign suggests that an increase in one factor corresponds to an increase in the response, whereas a negative sign indicates that a change in the factor results in a decrease in the response. Based on Eq (10), organic materials, inorganic materials, and bacteria had a significant effect on the Zn concentration of SRFs.

In comparing the three independent variables, the most important factor affecting the Zn concentration of SRFs was inorganic material. The highest Zn concentration of SRFs (93.48 mg g ^−1^) was related to run 3 (organic material: 0, inorganic material: LDH, inoculation bacteria with fertilizer: 1 W/V), which is 3.9-fold higher than the lowest run (24.54 mg g ^−1^) (run 9: organic material: pectin, inorganic material: 0, inoculation bacteria with fertilizer: 0). In comparing all runs, if biochar is used instead of pectin in SRFs, the Zn concentration increased by 42%. On the other hand, if pectin is combined with LDH or inoculated with *Bacillus subtilis*, the Zn concentration increased by 47% and 42%, respectively. This finding is consistent with the results of other researchers who stated that the release of elements from fertilizer is dependent on microorganisms. [25]. The order showing the influence of inorganic and organic types on Zn concentration was as follows: LDH > LDO and biochar > pectin, respectively. Additionally, the best bacterial ratio with SRFs was 2. In other words, the type of base is effective in releasing elements. [26,27]. Also, in another study, it was found that double-layer magnesium-aluminum hydroxide with a ratio of 3:1 was more successful in releasing elements compared to the ratio of 2:1, the ratio used in this experiment. It was also stated in this study that inoculation with growth-promoting bacteria improved the release of elements compared to non-inoculated compounds [28].

The univariate analysis of variance (ANOVA) was employed to assess the significance of differences among two or more factors, as well as to determine the adequacy of the model in fitting the predicted outcomes to the experimental data. In the context of response surface methodology (RSM) modeling, the model is considered appropriate and applicable when the p-value is below 0.05 (indicating significance) and the lack of fit is above 0.05 (indicating non-significance). In the present research, the result of ANOVA indicated that the model was suitable and usable because the p-value, lack of fit, F-value, R², adjusted R², predicted R², and Adeq-Precision were 0.0001, 0.324, 31.79, 0.95, 0.92, 0.75, and 21.52, respectively. The Adeq-Precision value represents the desired signal-to-noise ratio (S/N ratio), which should exceed 4. The Adeq-Precision values obtained from this research were adequate and desirable ratios. Thus, it can be concluded that the model had a high capability to predict Zn concentration of SRFs under different synthesis conditions.

### Interaction effects of variables using 3D response surface plots

**Fig 1:**
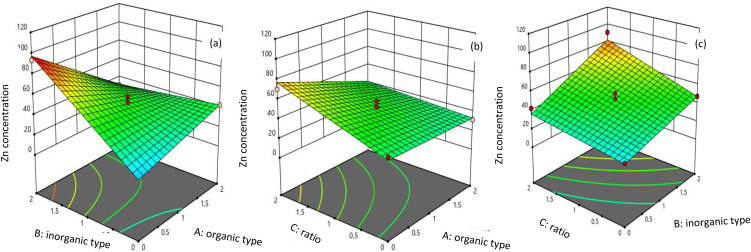
a-c. 3D interactive surface plot for Zn concentration under different synthesis condition, b, and represented interaction inorganic and organic type, interaction ratio and organic type, interaction ratio and inorganic type.

The diverse effects of the individual factors (organic type, inorganic type, and the inoculation of *Bacillus subtilis* with SRFs) and their interactions on the Zn concentration of SRFs can be effectively illustrated through the use of 3D response surface plots (Fig 1 a-c). As shown in Fig 1a, the response surface plots were constructed to illustrate the effect of organic and inorganic types on response at a constant ratio of *Bacillus subtilis* inoculant with SRFs (1 w/v). LDH increased Zn concentration by 75% and 50% compared to LDO and biochar, respectively.

Based on the results, biochar increased Zn concentration by 40% compared to pectin. Additionally, combining biochar with LDH increased Zn concentration by 60% compared to biochar alone. This result indicated that SRFs based on biochar were better than pectin, but their efficiency was lower than that of SRFs based on LDH. Consequently, to improve the efficiency of SRFs based on biochar, combining them with LDH is a good solution to enhance their effectiveness.

The response surface plots were constructed to show the effect of organic type and the ratio of Bacillus subtilis inoculant with SRFs, as illustrated in Fig 1b. According to this figure, it can be concluded that the best ratio was 2, and combining SRFs based on organic types with bacteria enhanced Zn concentration compared to SRFs based on organic types alone. Furthermore, there was not much difference between the SRFs based on inorganic types alone and their inoculation with bacteria; however, their combination increased Zn concentration by 50% (Fig 1c).

### Response surface optimization

RSM numerically optimized values for maximum Zn concentration are shown in Table 3. The three optimal conditions of factors for Zn concentration were selected and introduced as optimal SRFs for the following stages (Table 3). The first optimal condition for synthesizing SRFs for organic type, inorganic type, and bacterial inoculation ratio was 0, LDH, and 1 w/v, respectively. The second optimal condition was 0, LDO, and 2 w/v for organic type, inorganic type, and bacterial ratio, respectively, while the third optimal condition was biochar, LDO, and 2 w/v.

**Table 3:** Response surface methodology (RSM) results and optimal conditions for synthesizing slow release fertilizers (SRFs).

	Organic type	Inorganic type	ratio	Zn concentration	Desirability
1	0	LDH*	0	94.497	1.000
2	0	LDO**	2	94.131	1.000
3	biochar	LDO	2	103.367	1.000

*layer double hydroxide (LDH), **layer double oxide (LDO).

### Characterization of optimal slow release fertilizers

**Fig 2:**
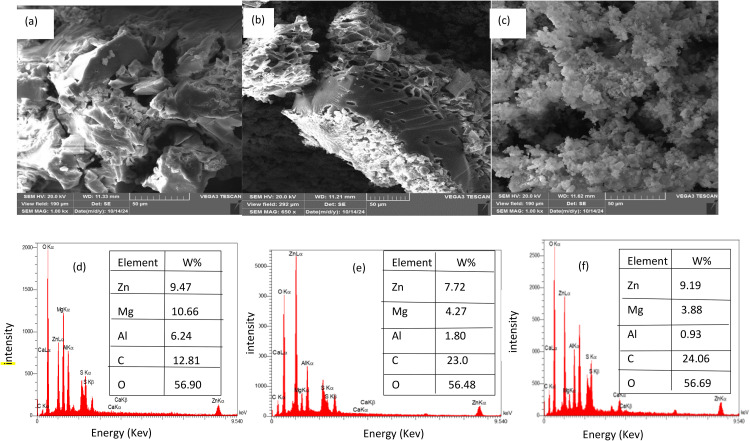
(a) SEM of Slow Release Fertilizer (SRF)1; (b) SEM of SRF2; (c) SEM of SRF3; (d) EDX of SRF1; (e) EDX of SRF2; (f) EDX of SRF3.

**Fig 3:**
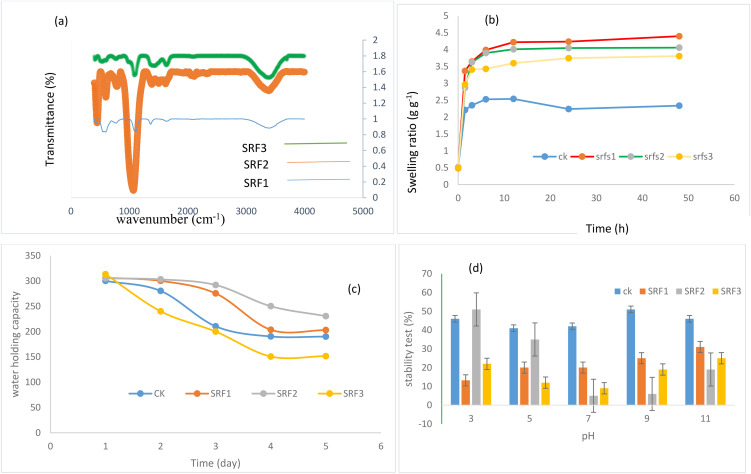
(a) FTIR of slow release fertilizers (SRFs); (b) swelling ratio of SRFs; (c) Water holding capacity of SRFs; (d) stability test (%) of SRFs.

The structures and morphologies of the three optimal SRFs (SRF-LDH (a), SRF-LDO-bacterial (b), and SRF-biochar-LDO-bacterial (c)) were evaluated. The results of the SEM obtained from the optimal SRFs exhibited a 3-D porous network structure in Fig 2 (a-c). These images show that after the adsorption process, the surfaces of all three fertilizers become rough and irregular. This phenomenon indicates that Zn has been well adsorbed onto the SRFs and has changed their surfaces. On the other hand, the EDX results (Fig 2 (d-f)) showed that the amount of magnesium (Mg) was higher than that of aluminum (Al), which is due to the 4:1 cation ratio of LDO and LDH. Additionally, the percentage of Zn in all three SRFs is almost consistent with Table 2. Furthermore, the temperature increased the specific surface area (SSA) of LDO by 24.4 times compared to LDH, facilitating the exposure of more active sites and thus generating greater access for the penetration of ions. The SSA of SRF-LDH, SRF-LDO-bacterial, and SRF-biochar-LDO-bacterial were 3.61, 73.25, and 65 m² g-¹, respectively. The FTIR spectra of SRF-LDH, SRF-LDO-bacterial, and SRF-biochar-LDO-bacterial are illustrated in Fig 3a. In the analyzed spectra, a prominent absorption peak is observed at 3000.72 cm-¹ and 3300.28 cm-¹, which is attributed to the stretching vibration of the -OH group as well as the intermolecular and intramolecular hydrogen bonds present in SRF-LDH, SRF-LDO-bacterial, and SRF-biochar-LDO-bacterial [28]. Furthermore, the stretching absorption peak of the -OH group exhibited a noticeable shift from 3261.72 to 3256.28 cm-¹ upon the addition of biochar, likely indicating the establishment of strong hydrogen bonds between biochar and LDH/LDO [29].

### Evaluation of swelling ratio, water-holding capacities and stability test

#### Swelling ratio.

In SRFs, the release of nutrients primarily occurs as water permeates through the coating layer into the core of the fertilizer. Consequently, the swelling characteristics of the SRFs are crucial for the gradual release of nutrients. To examine this phenomenon, the swelling behaviors of SRF1, SRF2, and SRF3 were evaluated in aqueous solutions at various time intervals (0, 1.5, 3, 6, 12, 24, 48 hours), as illustrated in Fig 3b. It is evident that all SRFs exhibit rapid expansion during the first 6 hours of immersion in water, followed by a deceleration in the rate of swelling. After 12 hours, the swelling levels of these fertilizers stabilized, whereas ZnSO_4_, used as a control, reached equilibrium more quickly (within 90 minutes). The highest swelling ratio was related to SRF1 (4.4 g g -¹). The swelling ratio of SRF3 (LDO-biochar-bacterial) was 3.81 g g -¹, which was higher than that of ZnSO_4_ (2.41 g g -¹). This result could be attributed to changes in the degree of hydrogen bonding resulting from the presence of biochar [5]. Notably, SRF2 also swelled 42% more than the control. This response is related to the hydrophilic properties and the higher number of active sites compared to zinc sulfate fertilizer. The swelling ratios obtained in this research were higher than those of other researchers. The swelling kinetics exhibited a strong linear relationship with both the pseudo-second-order (PSO) and Korsmeyer-Peppas models. The calculated Qe for the PSO model aligned well with the measured ESR values presented in Table 4. This suggests that the relaxation of molecular segments is a key factor governing the swelling process. Furthermore, the PSO model displayed superior R² values and correlation compared to the Korsmeyer-Peppas model, indicating that internal moisture diffusion plays a crucial role during the drying process of the fertilizer. Consequently, an increased water content results in a reduced drying rate [10]. Specifically, SRF1 demonstrates a rapid water absorption rate and a slower drying rate, whereas ZnSO_4_ dries more quickly. In summary, it can be concluded that the structure of SRF1 effectively mitigates the rapid release of Zn. Additionally, it provides significant hardness, which prevents the premature release of Zn due to particle breakage during transportation and application, thereby improving its agricultural utility.

**Table 4:** Swelling kinetic parameters for SRFs.

sample	Korsmeyer-Peppas model			Pseudo-second-order kinetic model			
	R2	N	k1	R2	Qe (g/g)	**ESR	k2 [g/(g·min)]
CK*	0.9555	0.04	2.21	0.9994	2.58	5.77	0.38
SRF***1	0.9802	0.07	3.36	0.9994	4.4	6.86	0.22
SRF2	0.9274	0.08	3.09	0.9998	4.06	4.42	0.24
SRF3	0.9509	0.06	3	0.9999	3.81	6.95	0.26

*: Control (ZnSO_4_); **: equilibrium swelling rate.***slow release fertilizers

### Water-holding capacities

The highest and lowest water – holding capacities were observed for SRF1 and ZnSO_4_, respectively. The absorption capacity of each fertilizer decreased over time, but this decrease was greater for ZnSO_4_ than for the other fertilizers. On the other hand, there was no significant difference between the absorption capacities of the three SRFs. After 5 days, the absorption capacity of ZnSO_4_ decreased by 51%, while that of SRF1 decreased by 20% (Fig 3c). This result shows that this fertilizer has the ability to retain water and can be used in arid and semi-arid areas that urgently need water.

### Stability test

One of the important characteristics of fertilizer is its stability at different pH levels. In this regard, the stability test was evaluated at different pH values (3 –11). This figure showed that the highest weight loss was related to ZnSO_4_ at all pH levels, while each fertilizer displayed a different response at varying pH levels. On the other hand, SRF1 was stable at all pH levels, changing less than the other fertilizers. In general, these results demonstrated that each fertilizer is suitable for a specific pH; for example, SRF3 was more stable at pH 5 than at pH levels above 7, while SRF2 was more stable at alkaline pH levels than at acidic ones. Therefore, it can be concluded that fertilizer SRF3 is suitable for acidic soils and SRF2 is suitable for alkaline soils (Fig 3d).

### Zn release performance and kinetic parameters

**Fig 4:**
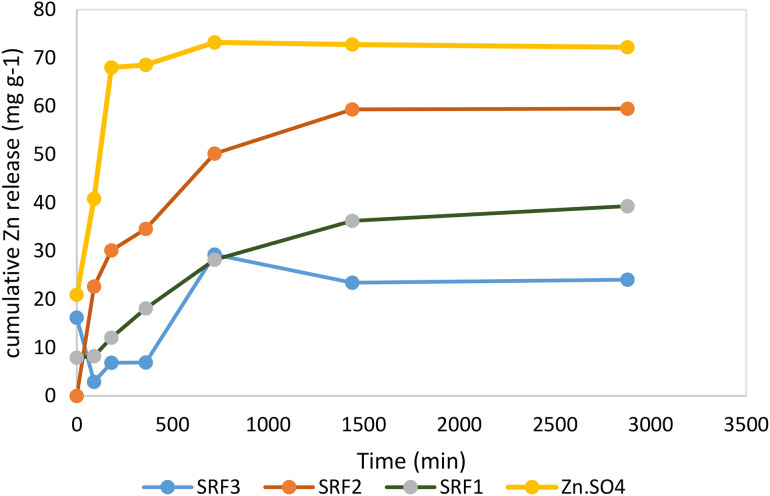
Cumulative Zn release from slow release fertilizers (SRFs) and ZnSO_4_.

The release of Zn from SRFs and ZnSO_4_ was evaluated at different times over 48 h, as shown in Fig 4. SRFs released Zn rapidly during the initial period of slow release (0–12 h) and then began to slowly release the remaining nutrients (12–48 h), while ZnSO_4_ released Zn rapidly (0–1.5 h) and then reached equilibrium. The slow-release phases of zinc from slow-release fertilizers (SRFs) can generally be divided into three distinct stages: the lag phase, the steady-state release phase, and the degradation phase. In the lag phase, the release of fertilizer is minimal as water gradually infiltrates the coating layer, with the swelling characteristics of the films being particularly significant at this stage. As illustrated in Fig 4 (SRF3), incorporating biochar into low-density polyethylene (LDO) coatings can diminish the swelling properties of the films, thereby reducing the duration of this phase. This observation aligns with the findings of [30], who noted that the inclusion of biochar can modify the hydrophilic properties of polymeric films, influencing their capacity for water absorption. During the steady-state release phase, increased water penetration into the coated fertilizer leads to the dissolution of some solid fertilizer, which subsequently releases nutrients through fissures in the swollen layer [31]. The release rate remains stable due to osmotic pressure differentials between the outer and inner regions of the coated fertilizer, a phenomenon extensively documented in existing literature [32]. Initially, the zinc release from SRF2 exceeded that of SRF1 and SRF3. Over the first 12 hours, SRF1, SRF2, and SRF3 released 56%, 45%, and 42% of zinc, respectively, while ZnSO_4_ released 72% of its nutrients, indicating that the incremental release from SRFs was less than that from ZnSO_4_. After 48 hours, the zinc release from SRFs was also lower than that from ZnSO_4_, suggesting that SRFs with larger particle sizes may impede the release of Zn. In contrast, when ZnSO_4_ was introduced to water, it dissolved and diffused rapidly.

Furthermore, the zero-order, first-order kinetic, Higuchi, and Peppas kinetic models were employed to analyze the Zn slow-release profiles in the water of SRFs, aiming to further elucidate the mechanisms of Zn release. The fitted equations, along with their R² and standard error (SE) values, are presented in Table 5. In the case of SRF1, the zero-order kinetic model exhibited the lowest R² value, followed by the R² of the Higuchi kinetic model, indicating that the slow-release process of SRF1 was non-linear and did not conform to the skeleton diffusion mechanism. Conversely, for SRF2 and SRF3, the R² value of the Peppas kinetic model surpassed that of the Higuchi model. Notably, in SRF1, the first-order kinetic model demonstrated a higher R² than the Peppas model, suggesting distinct nutrient release mechanisms between SRF1 and the other two SRFs. The Peppas kinetic model more accurately characterizes the slow-release behavior of SRF2 and SRF3. Additionally, a diffusion coefficient of 0.08 derived from the Peppas model indicates that SRF2 adheres to Fick’s law of diffusion

**Table 5:** Zn release kinetics parameters for slow release fertilizers (SRFs)).

	SRF1		SRF2		SRF3		Zn.SO_4_	
	R^2^	SE	R^2^	SE	R^2^	SE	R^2^	SE
First-order	0.90	0.45	0.97	0.10	0.95	0.19	0.65	0.18
Zero-order	0.61	0.88	0.83	0.95	0.83	0.75	0.42	0.12
Higuchi	0.88	0.43	0.54	0.34	0.52	0.32	0.32	0.17
Peppas	0.84	0.77	0.97	0.38	0.96	0.35	0.64	10.2

## Conclusion

In this study, the response surface optimization method was utilized to minimize costs and time. Ratios of organic (pectin, biochar), mineral (LDH, LDO), and bacterial inoculation with SRFs (1 and 2 w/v) were applied, leading to the development of 12 SRFs. Ultimately, modeling and optimization were performed. The findings showed that three fertilizers that met the desirability criteria (SRF-LDH (SRF1), SRF-LDO-bacterial (SRF2), and SRF-biochar-LDO-bacterial (SRF3)) were the most effective options. The research indicated that the fertilizer surfaces became rough and uneven following Zn adsorption, suggesting effective binding of Zn to the fertilizer surfaces. Moreover, the swelling percentage trait demonstrated that all three fertilizers exhibit greater swelling and retain moisture longer than the commercial ZnSO_4_. Conversely, the stability test findings showed that all fertilizers remained stable at various pH levels, with SRF2 proving effective for calcareous soil, while SRF3 was effective for acidic conditions. The slow-release behavior of Zn showed three phases in the gradual release of Zn from SRFs, including an initial phase of steady Zn release and a concluding phase of diminishing rates of slow release. The first-order and Higuchi kinetic models were more suitable for describing the nutrient slow-release mechanism of SRFs, suggesting that the surrounding environment can influence the gradual release of Zn from these fertilizers.
